# Activity disequilibrium between ^234^U and ^238^U isotopes in natural environment

**DOI:** 10.1007/s10967-014-3001-9

**Published:** 2014-02-27

**Authors:** Alicja Boryło, Bogdan Skwarzec

**Affiliations:** Department of Analytical and Environment Radiochemistry, Faculty of Chemistry, University of Gdańsk, Wita Stwosza 63, 80—308 Gdańsk, Poland

**Keywords:** Uranium isotopes, Activity disequilibrium, Southern Baltic, Environmental samples

## Abstract

The aim of this work was to calculate the values of the ^234^U/^238^U activity ratio in natural environment (water, sediments, Baltic organisms and marine birds from various regions of the southern Baltic Sea; river waters (the Vistula and the Oder River); plants and soils collected near phosphogypsum waste heap in Wiślinka (Northern Poland) and deer-like animals from Northern Poland. On the basis of the studies it was found that the most important processes of uranium geochemical migration in the southern Baltic Sea ecosystem are the sedimentation of suspended material and the vertical diffusion from the sediments into the bottom water. Considerable values of the ^234^U/^238^U are characterized for the Vistula and Oder Rivers and its tributaries. The values of the ^234^U/^238^U activity ratio in different tissues and organs of the Baltic organisms, sea birds and wild deer are varied. Such a large variation value of obtained activity ratios indicates different behavior of uranium isotopes in the tissues and organisms of sea birds and wild animals. This value shows that uranium isotopes can be disposed at a slower or faster rate. The values of the ^234^U/^238^U activity ratio in the analyzed plants, soils and mosses collected in the vicinity of phosphogypsum dumps in Wiślinka are close to one and indicate the phosphogypsum origin of the analyzed nuclides. Uranium isotopes ^234^U and ^238^U are not present in radioactive equilibrium in the aquatic environment, which indicates that their activities are not equal. The inverse relationship is observed in the terrestrial environment, where the value of the of the ^234^U/^238^U activity ratio really oscillates around unity.

## Introduction

Natural uranium consists of three alpha radioactive isotopes: 99.2745 % of ^238^U, 0.7200 % of ^235^U, and 0.0054 % of ^234^U [1, 2]. Uranium isotopes ^234^U and ^238^U are not present in radioactive equilibrium in the natural environment, which indicates that their activities are not equal. Especially in the aquatic environment deviations from the equilibrium are large. The average values of the activity ratio between ^234^U and ^238^U are in the range from 0.51 to 9.02 for groundwater, from 1.11 to 5.14 for salt water, from 1.00 to 2.14 for river water, from 0.80 to 1.00 for river suspension, 1.14 for oceanic water and 1.17 for Baltic water [3–7]. In rocks, soils and sediments uranium isotopes ^234^U and ^238^U are in relative equilibrium (from 0.84 to 1.19 for oceanic basalts, from 0.70 to 1.16 for phopshorite concretions, from 0.83 to 1.28 for oceanic sediments and from 0.98 to 1.04 for Baltic sediments) [8, 9]. There are several reasons for the radioactivity disequilibrium: radioactive decay energy, related to the secretion of α particles with nuclei of atoms, causes a “kickback” to the newly created isotopes for distance 10^−7^–10^−6^ cm from sites in the crystal lattice occupied by atoms of the isotope ^238^U output. Consequently, the ^234^U atoms are less related to the structure of minerals than the ^238^U atoms, and easier to diffuse to the surface of mineral grains and the cracks. In the oxidizing environment of the surface layer of minerals containing uranium from the water, ^234^U atoms are more easily leached into solution, yielding the oxidizing process to uranium(VI) faster than ^238^U atoms. Hence the isotope ^234^U shows greater mobility in the surface area. The differences in geochemical behavior between ^234^U and ^238^U isotopes are marked in surface environments, where the waters are more enriched in ^234^U in relation to ^238^U, while in the rocks inverse relationship is observed [10].

The principal source of uranium in the natural environment is the atmospheric precipitation of terrigenic material, soil resuspension, rock weathering, as well as river waters and fertilizers. Moreover, the concentration of uranium in the natural environment is increased by human activity including industry, fossil fuel combustion, phosphate fertilizers in agriculture, and domestic and industrial sewage. On the other hand, large amounts of uranium contents are produced by the modern industry: metallurgy, oil refinery, nuclear industry, nuclear weapon tests, the use of uranium ammunition, the manufacture and processing of fuel rods, ore mining, as well as phosphogypsum waste heaps [7, 11–22].

The aim of this work was estimation of the ^234^U/^238^U activity ratio in the samples from the southern Baltic and Northern Poland. The issue of the presence of uranium isotopes in the environment is very important because it is used to distinguish natural from anthropogenic uranium and provides information about possible high concentrations of uranium in the investigated sites determined by anthropogenic sources. The analysis of the activity of ^234^U/^238^U ratio values was applied to identify whether uranium isotopes were of natural or anthropogenic origin.

## Materials and methods

**Fig. 1:**
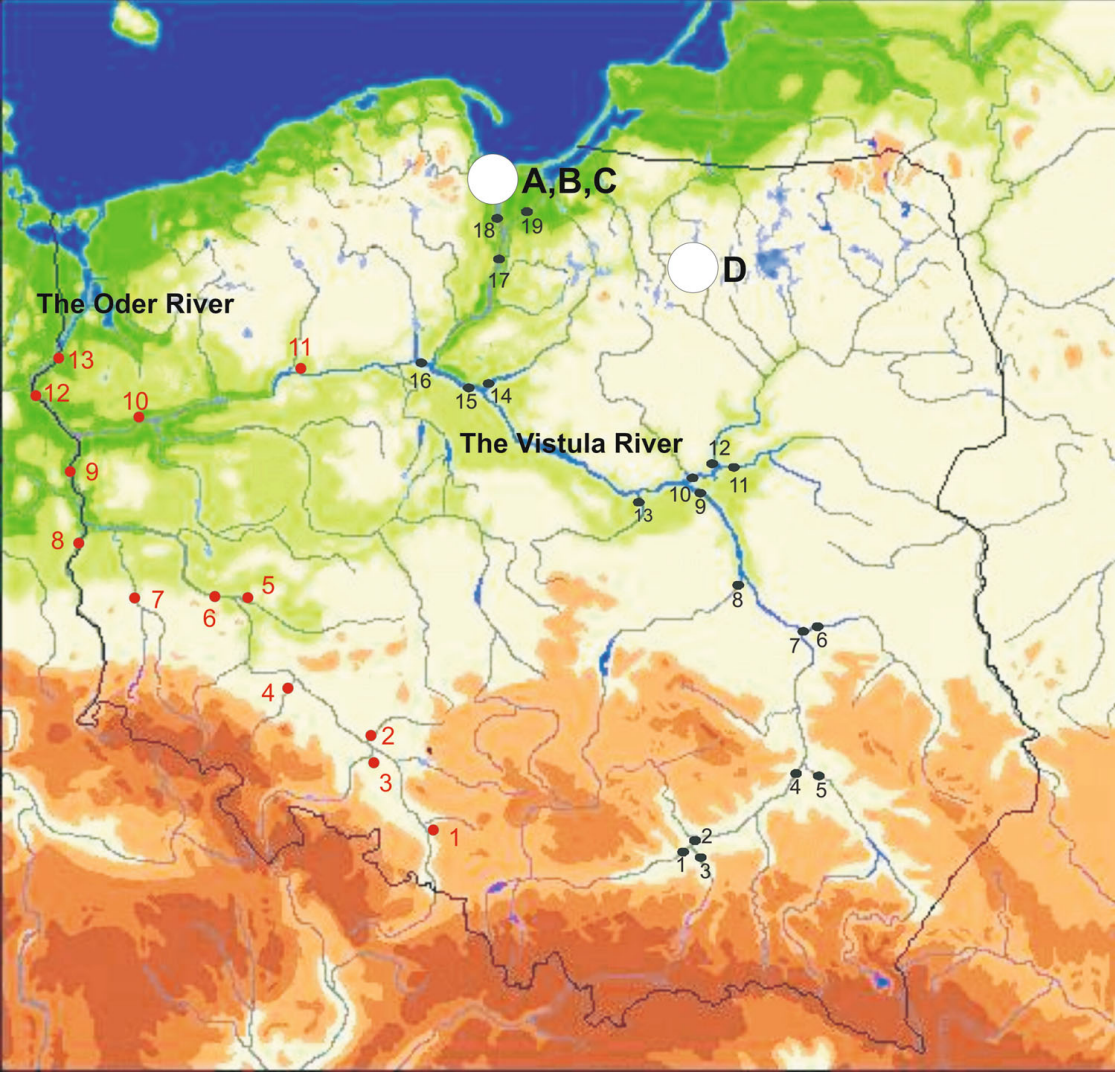
The places of sampling from the Northern Poland (*A*, *B*, *C* sea birds, mosses and phosphogypsum stockpile in Wiślinka, *D* deer, roe deer and fallow deer and the Vistula and Oder Rivers). Among the major tributaries of the Vistula: *1* the Vistula (Cracow), *2* the Nida, *3* the Dunajec, *4* the Vistula (Sandomierz), *5* the San, *6* the Wieprz, *7* the Vistula (Dęblin), *8* the Pilica, *9* the Vistula (Warsaw), *10* the Bug with the Narew, *11* the Bug, *12* the Narew, *13* the Bzura, *14* the Drwęca, *15* the Vistula (Toruń), *16* the Brda, *17* the Vistula (Grudziadz), *18* the Leniwka, *19* the Nogat. Among the major tributaries of the Oder: *1* the Oder (Chałupki), *2* the Mała Panew, *3* the Nysa Kłodzka, *4* the Bystrzyca, *5* the Barycz, *6* the Oder (Głogów), *7* the Bóbr, *8* the Nysa Łużycka, *9* the Oder (Słubice), *10* the Warta, *11* the Noteć, *12* the Oder (Gozdowice), *13* the Oder (Widuchowa)

The environmental samples were collected in the region of the Baltic Sea (water, sediments, Baltic organisms and marine birds), Northern Poland (kidney, liver and muscle tissue samples of deer, roe deer and fallow deer) and around phosphogypsum stockpile in Wiślinka in the years 1997–2009 (Fig. 1). The river water samples were collected seasonally (every 3 months) down the main course of the Vistula and the Oder and their major tributaries (Fig. 1).

The analytical procedure of uranium determination of uranium isotopes (^234^U, ^238^U) in analyzed samples was based on co-precipitation of water samples and mineralization in concentrated acids HNO_3_, HCl and HF, and separation on the anion exchange resins according to the procedure given by Skwarzec and Boryło [7, 23–25]. The activities of ^234^U and ^238^U were measured using an alpha spectrometer (Alpha Analyst S470). The concentrations of uranium isotopes in the IAEA-300, IAEA-367, IAEA-312 and IAEA-375 samples were consistent with the reference values reported by the IAEA. The minimum detectable activity (MDA) of uranium radionuclide is between 0.10 and 0.15 mBq.

## Results and discussion

**Fig. 2:**
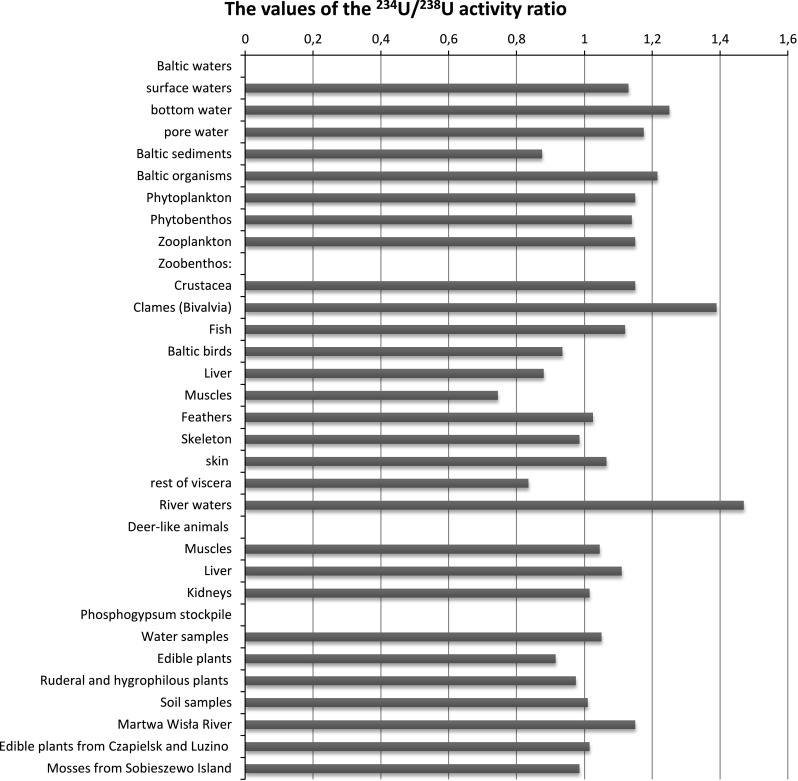
The values of the ^234^U/^238^U activity ratio in the analyzed components

The uranium content in the analyzed water samples and sediments of the southern Baltic was differentiated (Table 1; Fig. 2). In marine environment uranium exists principally as U(VI) and U(IV), where uranium(IV) compounds are weakly dissolved and in reduction areas the growth of stable form of uranium(IV) can be expected [26]. In neutral or little alkaline waters uranium(VI) exists predominantly in the dissolved carbonate anions [UO_2_(CO_3_)_3_]^4−^ and [UO_2_(CO_3_)_2_]^2−^ [27–29]. This autogenic uranium in seawater should be accumulated in marine organisms. The mean values of the ^234^U/^238^U activity ratio in ocean water are estimated at 1.14, while in the Baltic water 1.17 [7, 30].

**Table 1:** Average values of the ^234^U/^238^U activity ratio in environmental samples from the southern Baltic and Northern Poland

Component	The values of the ^234^U/^238^U activity ratio	References
Baltic waters		[31, 32]
Surface waters	1.12–1.14 ± 0.02	
Bottom water	1.16–1.34 ± 0.03	
Pore water	1.17–1.18 ± 0.02	
Baltic sediments	0.48–1.27 ± 0.05	[31, 32]
Baltic organisms	1.04–1.39 ± 0.04	[7, 33, 34]
Phytoplankton	1.15 ± 0.01	
Phytobenthos	1.14 ± 0.01	
Zooplankton	1.15 ± 0.01	
Zoobenthos		
*Crustacea*	1.15 ± 0.01	
Clams (*Bivalvia*)	1.39 ± 0.04	
Fish	1.12 ± 0.01	
Baltic birds	0.75–1.12 ± 0.03	[35]
Liver	0.69–1.07 ± 0.03	
Muscles	0.35–1.14 ± 0.02	
Feathers	0.90–1.15 ± 0.03	
Skeleton	0.72–1.25 ± 0.04	
Skin	0.96–1.17 ± 0.02	
Rest of viscera	0.55–1.12 ± 0.01	
River waters	1.00–1.94 ± 0.03	[36, 37]
Deer-like animals		[38]
Muscles	0.76–1.33 ± 0.01	
Liver	0.81–1.41 ± 0.02	
Kidneys	0.61–1.42 ± 0.03	
Phosphogypsum stockpile		
Water samples	1.00–1.10 ± 0.02	[16–20]
Edible plants	0.83–1.00 ± 0.03	[16–20]
Ruderal and hygrophilous plants	0.96–0.99 ± 0.03	[16–18, 20]
Soil samples	0.98–1.04 ± 0.03	[16–18, 20]
Martwa Wisła River	1.10–1.20 ± 0.04	[16–20]
Edible plants from Czapielsk and Luzino	0.99–1.04 ± 0.02	[16–20]
Mosses from Sobieszewo Island	0.97–1.00 ± 0.02	[43]

The values of the ^234^U/^238^U activity ratio in surface and bottom water are comparable for all the analyzed basins and range from 1.12 ± 0.03 to 1.19 ± 0.02 in the Bornholm Deep, from 1.14 ± 0.01 to 1.18 ± 0.02 in the Gdańsk Deep, and from 1.13 ± 0.03 to 1.19 ± 0.03 for the Słupsk Narrow [31] and are typical for groundwater, where the values of this activity ratio are between 0.51 and 9.02. The obtained results of activity ratio values in the bottom water samples are therefore comparable to the value characteristic of the Baltic waters. The values of the activity ratio in the pore water samples from the southern Baltic ranged from 1.17 ± 0.03 to 1.18 ± 0.03 and are consistent with the value recorded for the Baltic waters (Table 1; Fig. 2) [31, 32]. This does not mean that the uranium contained here is of hydrogenic origin, because its concentration in the pore water is significantly higher than in the bottom water. Activation of uranium to the pore water is possible due to the processes taking place in diagenetic sediment material, due to the release of adsorbed uranium by organic matter [31, 32].

The values of the activity ratio between ^234^U and ^238^U uranium isotopes in sediments from the southern Baltic increase with depth of the sediment core, indicating the vertical diffusion processes of uranium from sediments to the bottom water through the pore water, and diagenetic changes occurring in the sediment material. The pore water plays a very important role in the uranium migration because it penetrates the superficial layers of sediments and increases values of the ^234^U/^238^U activity ratio, as well as causes leaching of uranium from the sediment into the sea water. The specific effect of the pore water is made clear in the sediments from the Słupsk Bank, where the ^234^U/^238^U activity ratio ranged from 0.48 ± 0.01 to 0.98 ± 0.03. The values of the ^234^U/^238^U activity ratio in the other analyzed sediments of the southern Baltic are in the range between 0.82 ± 0.04 and 1.27 ± 0.04 and are close to the values of the Baltic sediments (0.98–1.04) [32]. The values of ^234^U/^238^U activity in the bottom sediments of Słupsk Bank are close to the values in the nodules (0.78–1.36 in nodules and 0.70–1.16 in phosphoric nodules). The increase of the activity ratio in the upper layers of sediments is associated with the effect of terrigenic material falling. On the other hand, this layer is under significant influence of sea water, which causes a clear increase in the ^234^U/^238^U activity ratio in the surface layer of sediment as compared with segments lying below the sediment layer. Uranium nuclides (^234^U and ^238^U) in the sediments of the Gdansk Basin, the Słupsk Narrow and the Bornholm Deep are in relative equilibrium because the values of the ^234^U/^238^U activity ratio ranged from 0.92 to 1.06 [31]. The high values of the ^234^U/^238^U activity ratio were observed in sediments and surface waters of the Gdańsk Deep only after the flood that passed through Poland in 1998. Water flowing into Gdańsk Bay mainly from the Vistula River, introduced a significant part of nutrient salts, which contain big amounts of anthropogenic uranium. Thus, the concentration of uranium in surface waters is determined by the proportion in which the waters of the Baltic Sea and river waters are mixed. The analysis of sediment samples, surface and bottom waters of the southern Baltic Sea shows a special role of the fresh waters, because after the flood in 1997 in all the analyzed samples, the concentration of uranium and values of the ^234^U/^238^U activity ratio were higher (1.34 ± 0.01 for the bottom waters and 1.27 ± 0.04 for sediments) (Table 1; Fig. 2). The high values of the ^234^U/^238^U activity ratio in the basin of the Gdańsk Deep are associated with the presence of uranium from the phosphate fertilizers in cultivated fields and uranium contained in the salty waters of mines. Into the bottom sediments in the Gdańsk Deep reservoir flow river waters, which contain not only uranium from the weathering of rock, but also anthropogenic uranium associated with human activity, carried out mainly in agriculture and mining in the catchment area of the Vistula [31, 32].

The distribution of radionuclides, including uranium, in aquatic organisms is a very difficult process. This is mainly due to the great diversity of many species of aquatic organisms which are involved in a complicated series of aquatic food chains. In addition, higher organisms, as opposed to lower, due to the possession of various organs, have a more uneven accumulation of radionuclides. Knowledge of the uranium content in the tissues of marine organisms and in the surrounding water allows to assess the degree of its accumulation in biota. This value is referred to as the coefficient of concentration of the element. Baltic organisms are characterized by very low affinity for uranium, in contrast to the polonium and plutonium. The source of uranium in the Baltic plants and animals is the sea water (Table 1; Fig. 2). The values of the ^234^U/^238^U activity ratio in the representatives of the phytoplankton and zooplankton from the southern Baltic (from 1.13 to 1.16) are very similar to the values in ocean water and Baltic water (1.14 and 1.17 respectively) [33, 34]. The values of the ^234^U/^238^U activity ratio in zoobenthos organisms ranged from 1.04 to 1.39. The lowest values of the ^234^U/^238^U activity ratio were estimated for fish from the Baltic (1.10–1.13). The average value of the ^234^U/^238^U activity ratio in the analyzed Baltic organisms is 1.15 and is close to the values, which characterize the Baltic seawater (1.18 and 1.17, respectively). This fact proves that the main source of uranium in the Baltic organisms is sea water [7, 33, 34].

Structure and physiology of birds is constantly changing during the successive phases of growth, as well as under the influence of several physico-chemical factors in the environment. The accumulation of uranium in their bodies is related primarily to the content of the element in the diet, the concentration of radionuclides in the environment in which birds live. Seabirds are typical animals, which live both in water and on land, and to their body penetrate both radionuclides from the marine waters and from the air. Uranium isotopes were determined in the marine birds from the Polish area of the southern Baltic Sea among species permanently residing at the southern Baltic (razorbill *Alca tarda*, great cormorant *Phalacrocorax carbo*, eurasian coot *Fulica atra*), species of wintering birds (tufted duck *Aythya fuligula*, common eider *Somateria* *mollissima,* long-tailed duck *Clangula hyemalis,* velvet scoter *Melanitta fusca*) and species of migrating birds (black guillemot *Cepphus grylle*, red-throated diver *Gavia stellata*, common guillemot *Uria aalge*)). The average values of the ^234^U/^238^U activity ratio in whole organisms of analyzed marine birds lie between 0.75 ± 0.02 in black guillemot (*C. grylle*) and 1.12 ± 0.03 in common eider *(S. mollissima*) (Table 1; Fig. 2) [35]. The slightly lower values were measured in phytoplankton and zooplankton organisms and in fishes from the southern part of the Baltic Sea [7]. The values of ^234^U/^238^U activity ratio in tissues and organs of analyzed marine birds range widely between 0.35 ± 0.02 in muscles of black guillemot (*C. grylle*) and 1.25 ± 0.05 in skeleton of red-throated diver (*G. stellata*), but for most of analyzed species lie around 1. The lowest values of ^234^U/^238^U activity ratio were observed in rest of viscera (from 0.55 ± 0.02 to 1.12 ± 0.03) and muscles (from 0.35 ± 0.02 to 1.14 ± 0.07), whereas the highest in skeleton (from 0.72 ± 0.06 to 1.25 ± 0.05) [35]. These results show different behavior of uranium isotopes in sea birds organisms and fast or slow speed of uranium isotopes disposal from different organs and tissues of sea birds. Among all analyzed samples only in the marine birds samples the estimation of ^235^U content was successful. Therefore, the ^235^U/^238^U activity ratio is discussed in the further part of this paper. The values of the ^235^U/^238^U activity ratio in the analyzed organs and tissues of marine birds range from 0.032 ± 0.003 in skeleton of common eider to 0.050 ± 0.008 in feathers of great cormorant, however in the majority of cases were between 0.033 ± 0.004 and 0.041 ± 0.005. The obtained results are in agreement to values reported for marine organisms in southern Baltic Sea [7].

Among the many sources of radioactive isotopes, which constitute direct or indirect threat for the rivers and the Baltic Sea, the most important are fallout, coal mines and sewage discharged from nuclear power plants. Analyzed concentrations of uranium isotopes ^234^U and ^238^U were very diverse. Higher uranium concentrations were found in the basin of the Vistula and Oder Rivers in the spring and the autumn, while the smallest in the summer. The values of the ^234^U/^238^U activity ratio in river waters range from 1.22 to 1.40 (the average 1.31) [7].

Uranium isotopes ^234^U and ^238^U are not in radioactive equilibrium in the Vistula and Oder Rivers water samples and values of this proportion are between 1.00 and 2.14 (Table 1; Fig. 2). The greatest values of the ^234^U/^238^U activity ratio along the main stream of the Vistula and Oder Rivers were observed in the summer in Kraków (1.95 ± 0.15) and Głogów (1.84 ± 0.06) respectively, the lowest in the winter in Malbork (1.05 ± 0.03) and Gozdowice (1.20 ± 0.06) (respectively) [36, 37]. Among the Vistula and Odra tributaries the largest value of the ^234^U/^238^U activity ratio was recorded in the waters of the Bzura (1.62 ± 0.15) and the Bystrzyca (1.61 ± 0.03), the smallest in the Bug with the Narew (1.02 ± 0.08) and the Noteć (1.03 ± 0.06) respectively [36, 37].

In the Vistula and Oder Rivers, with the increase of salinity, the values of the ^234^U/^238^U activity ratio decreases. Greater differentiation of the ^234^U/^238^U activity ratio was observed in the spring and the autumn, which is associated with the use of phosphate fertilizers in agriculture, increased underground and surface runoff of snowmelt water, the discharge of saline mine waters from the Upper Silesian Industrial Region and Lower Silesian Coal Basin, increased deposition of dry atmospheric fallout in the winter period—the burning of coal, oil and gas and increased soil erosion and leaching of substances from the soil by infiltrating water and transporting uranium with the material of the river. The higher values of the concentration of uranium and ^234^U/^238^U activity ratio were calculated for the mountain Polish rivers in contrast to the plain Polish rivers. A particularly high values of the ^234^U/^238^U activity ratio were observed in the Bystrzyca (from 1.44 ± 0.04 in summer to 1.61 ± 0.03 in autumn) [37]. The Bystrzyca is a typical mountain river and therefore a higher concentration of uranium in river water is mainly a result of higher concentrations of this element in the parent’s rocks. The tributary of the Bystrzyca basin is in the Karkonosze Mountains, which are built mainly from gneiss, pink and red granite, where the uranium concentration reaches about 20 g/ton and slate with lots of crystalline dolomite and marble. In aquatic environment uranium is mobile in oxidizing conditions and is leached from the rocks to surface waters. The source of ^238^U in the Bystrzyca is not only the leaching of uranium, but also the presence of this nuclide in the salty waters of the mine (Lower Silesian Coal Basin). The value of the ^234^U/^238^U activity ratio in salty water ranges from 1.11 to 5.14. The values of the ^234^U/^238^U activity ratio in the Bystrzyca are also close to the values, which are in mining drilling (1.55). The large values of the analyzed activity ratio are caused by human activities in the basin, mainly in agriculture (using phosphate fertilizers) and mining (mine water discharge into the river) as well as the presence of uranium ores in these areas.

The values of ^234^U/^238^U activity ratio in the deer-like animals of Northern Poland were 0.61 ± 0.08–1.42 ± 0.28 for kidney, between 0.81 ± 0.21–1.41 ± 0.26 for liver and 0.76 ± 0.07–1.33 ± 0.18 for muscle (Table 1; Fig. 2) [38]. For the remaining samples the values were generally close to 1. For comparison, ^234^U/^238^U activity ratio in mushrooms from Poland is close to 1 [39] and in the various food products from the Wałbrzych region 1.16 ± 0.21 [40]. The obtained results of activity ratio for deer animals are close to the values obtained for marine birds and representatives organisms of the Baltic Sea. The varied ratios between ^234^U and ^238^U for deer animals may be associated with various species analyzed diet [38].

The ^234^U/^238^U activity ratio is approximately one in the phosphogypsum samples, and was estimated at 0.90 and 0.97. The analysis of values of the ^234^U/^238^U activity ratio in water samples from the Martwa Wisła River indicated that uranium is eluted from phosphogypsum waste and via river system is transported to the Bay of Gdańsk [17]. The low values of the ^234^U/^238^U activity ratio in analyzed water samples taken in the vicinity of the phosphogypsum waste heap indicate that uranium is lixiviated from phosphogypsum waste dump to retention reservoir and pumping station. Isotopes of uranium in the bottom water of the analyzed retention reservoir and pumping station around the phosphogypsum stockpile increase with depth, indicating the diffusion process from dump surface to water. This way of uranium elimination causes the increase of the activity ^234^U/^238^U ratio in surface water. The high values of the ^234^U/^238^U activity ratio in surface water samples taken from the Martwa Wisła River show that the migration and distribution of uranium radionuclides from the phosphogypsum waste heap to the Martwa Wisła River are rather slow. The values of the activity ratio in waters from the Martwa Wisła river range from 1.03 ± 0.07 to 1.17 ± 0.06 and are lower than characteristic values for fresh water of precipitation origin [18]. We observed that the values of the ^234^U/^238^U activity ratio in the water with immediate surroundings of waste heap were close to 1, while the in surface river water from the Martwa Wisła River were higher than one. The values of the activity ratio in waters of retention reservoir are typical of soil and rock samples [18, 20].

The values of ^234^U/^238^U activity ratio for vegetables which were collected around phosphogypsum stockpile in Wiślinka (Northern Poland) are between 0.83 ± 0.07 to 1.00 ± 0.05 (Tab. 1, Fig. 2) [18, 20]. It is a typical feature of the phosphoric rocks used for fertilizer production or typical of phosphogypsum samples. The atmospheric origin of uranium content depends not only on falling velocity from the atmosphere, but also on the age of leaves and their surface area and weather. These radionuclides fall to the plants together with aerosol particles by washout and sedimentation. It is typical feature of the phosphoric rocks used for fertilizer production, where these values ranged between 1.00 and 1.11 [15] or typical of phosphogypsum samples. The similar result was also noticed in an estuarine system in southwest Spain. The authors suggest that the equilibrium between ^234^U and ^238^U found in the Odiel river is of an external origin for the suspended matter particles, probably from the fertilizer complex [41, 42]. The ratios of ^234^U/^238^U in analyzed plant species which were collected from Czapielsk (29 km from the phosphogypsum waste heap, near Kartuzy city) and Luzino (55 km from the phosphogypsum waste heap, near Wejherowo city) are from 0.99 ± 0.06 to 1.04 ± 0.11 (Table 1; Fig. 2) [18, 20]. The values of the activity ^234^U/^238^U ratio in ruderal and hygrophilous plants around phosphogypsum stockpile ranged from 0.96 ± 0.06 to 0.97 ± 0.12 and from 0.98 ± 0.06 to 0.99 ± 0.04 respectively and indicate that uranium in analyzed plants originates from the phosphogypsum waste heap [18]. It is particularly important that the highest values of the ^234^U/^238^U activity ratio were characterized for ruderal plants, which are covered with tomentose hairs. The main source of isotopes of uranium is atmospheric fallout in the immediate vicinity of the phosphogypsum waste heap. Apart from atmospheric fallout, the plants can receive radionuclides from radioactive fallout and from the fertilizer added to phosphogypsum soil. The values of the ^234^U/^238^U activity ratio in soils depends not only upon the uranium concentration in the soil, but also on the amount of irrigation applied. The values of the ^234^U/^238^U activity ratio in samples of soils around the phoshpogypsum stockpile ranged from 0.98 ± 0.06 to 1.04 ± 0.05 (Table 1; Fig. 2) [18]. Atmospheric deposition is therefore the main source of uranium in the soil samples and the incorporation of the radionuclide occurs mainly from the wet deposition (rainfall) [18].

**Fig. 3:**
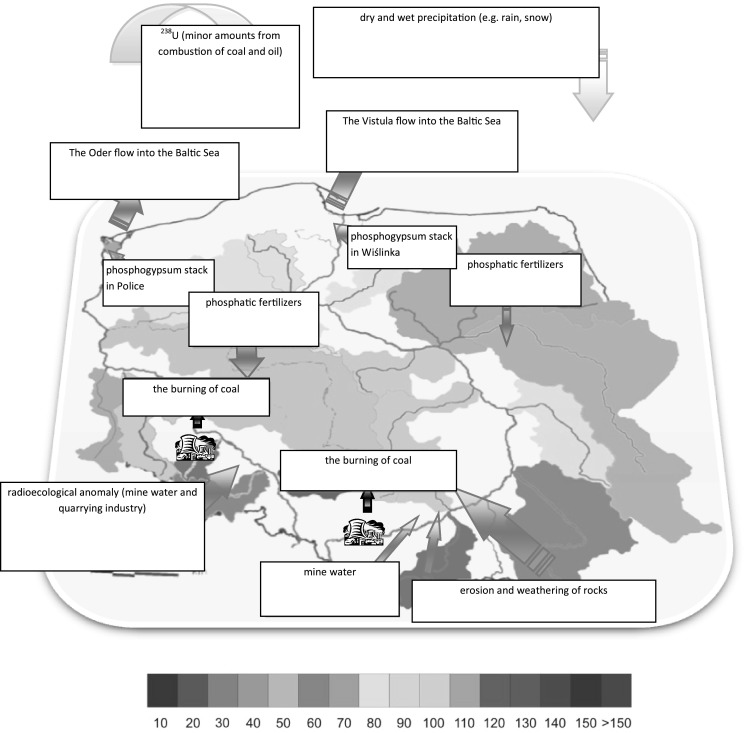
The uranium sources in the Baltic Sea from the basin of the Vistula River and the Oder River

The values of the ^234^U/^238^U activity ratio in analyzed mosses from Sobieszewo Island range from 0.97 ± 0.03 to 1.00 ± 0.07 and indicate that uranium in analyzed species originates from the phosphogypsum waste heap in Wiślinka [43]. The area around Wiślinka village is strongly polluted by uranium origin from phosphogypsum [16–18]. A rich and extensive as well as thick and dense turfs of mosses may store high amounts of uranium, which are deposited from atmosphere by winds or rains. In these plants, the accumulation degree is much higher than in vascular plants growing in the same habitats and depends on the structure of most the sod (thickness or density) [44]. Any differences between the obtained values of the ^234^U/^238^U activity ratio concentrations of uranium are probably related with location and distance from potential sources of contamination. The potential sources of uranium contamination in the Baltic Sea from Poland, which were defined in this article are presented on the Fig. 3. The main sources of uranium in Northern Poland, which significantly affect the value of the activity ratio between ^234^U and ^238^U in both terrestrial as well as marine environment are phosphogypsum stockpiles, located on the territory of Poland in Police and Wiślinka near Gdańsk. Police is a town in the West Pomeranian Voivodeship, northwestern Poland. Police town is situated on the Oder River and its estuary, south of the Szczecin Lagoon and the Bay of Pomerania. In central Poland large amounts of uranium are the result of the use of phosphate fertilizers many by farms. The largest producers of phosphate fertilizers in Poland are currently Chemical Works Police SA and Gdansk Phosphate Fertilizer Plants. Uranium sources in southern Poland are burning of coal and coal mining. Coal is a strategic resource in Poland, it meets 60 % of the energy needs of the country. Polish resources of this rock are among the largest in the world. Most of them are located in the Lublin province and Upper Silesia. Lublin resources are greater, lie on a smaller depth and are of high quality, although they are less well documented. The deposits extend from Łuków and Radzyń Podlaski to Hrubieszów and combine with the Lvov-Volyn Coal Basin, Ukraine. For many years Poland took top place among five countries with the largest coal mining. Another important source of uranium discovered in Upper Silesia is the so-called radioekological anomaly, resulting from mine water and quarrying industry. This phenomenon has been discussed on the example of the Bystrzyca River.

Small amounts of uranium are the results erosion and weathering of rocks, minor amounts from combustion of coal and oil and dry and wet precipitation (e.g. rain, snow).

## Conclusion

The obtained values of the ^234^U/^238^U activity ratio in all analyzed plants and soils near phosphogypsum stockpile in Wiślinka (Northern Poland) are close to one, which indicates that source of uranium in analyzed plants is phosphogypsum. The values of the ^234^U/^238^U activity ratio in water with immediate area of waste heap are considerably lower than in the waters of the Martwa Wisła river. The values of the ^234^U/^238^U activity ratio are approximately about one in the phosphogypsum and in the water of retention reservoir and pumping station, while in the water from the Martwa Wisła river they are slightly higher than one. Also the influence of phosphogypsum on radioactive contamination of environmental zone around the heap waste in Wiślinka (Northern Poland) was observed for mosses from Sobieszewo Island.

The obtained values of the ^234^U/^238^U activity ratio in surface, bottom and pore water samples from the southern Baltic were similar to those characteristic of sea water. Increased values of the activity ratio between ^234^U and ^238^U isotopes were found only in sea water and sediments that were collected for analysis during heavy and prolonged rains. Rivers flowing into the Gulf of Gdansk, mainly the Wisła River, introduce nutrient salts containing increased amounts of anthropogenic uranium. The obtained studies showed that the sources of uranium isotopes in Baltic water are inflows from the Vistula and Oder Rivers. The value of the activity ratio decreases over the Vistula and the Oder River from its source to its mouth, which involves changing the salinity of the river. The values of the ^234^U/^238^U activity ratio in surface marine waters are designated by proportion of mingling of the Baltic waters with river waters. The values of the ^234^U/^238^U activity ratio in the sediments of the southern Baltic decrease with the sediment depth, which suggests its diffusion from bottom sediments to the bottom water through pore waters and diagenetic transformations in the sedimentary material. The content of uranium in the sediments of the southern Baltic is a result of its penetration into the sediments profile and its flow with pore (e.g. Ławica Słupska). In the other regions of the southern Baltic uranium isotopes are in a relative equilibrium, which is reflected by the ^234^U/^238^U activity ratio ranging between 0.99 and 1.10. The average value of the ^234^U/^238^U activity ratio in the analyzed Baltic organisms and marine birds is close to the value, which characterizes the Baltic seawater which suggests that the main source of uranium in these organisms is sea water. Significant differences of values of the ^234^U/^238^U activity ratios were observed in the various tissues and organs of examined birds and Baltic organisms. Average values of the ratio of activity between ^234^U and ^238^U in individual tissues of deer-like animals fluctuated around unity and were slightly lower than calculated for organisms and fish that inhabit the southern part of the Baltic Sea.

## References

[1] Delacroix D, Guerre JP, Leblanc P, Hickman C (1998). Radionuclide and radiation protection. Data handbook.

[2] Browne E, Firestone FB, Shirley VS (1986). Table of radioactive isotopes.

[3] Baturin GN (1975) Atomizdatat 152

[4] Ku TL, Knauss KG, Mathieu GG (1977). Deep Sea Res.

[5] Baar G, Lambert SJ, Carter JA (1979). Isot Hydrol.

[6] Szefer P (1987). Stud Mater Oceanol.

[7] Skwarzec B (1997). Ambio.

[8] Szefer P (1987). Stud Mater Oceanol.

[9] Szefer P (1987). Stud Mater Oceanol.

[10] Fleischer RL, Raabe OG (1978). Geochim Cosmochim Acta.

[11] Bolivar R, García-Tenorio M, García-León J (1996). J Radioanal Nucl Chem.

[12] Martinez-Aguirre A, Garcia-León M (1997). J Environ Radioact.

[13] Martinez-Aguirre A, Garcia-Orellana I, Garcia-León M (1997). J Environ Radioact.

[14] Vrecek P, Benedik L (2002). Mine Water Environ.

[15] Sam AK, Holm E (1995). Sci Total Environ.

[16] Skwarzec B, Boryło A, Kosińska A, Radzajewska S (2010). Nukl.

[17] Boryło A, Nowicki W, Skwarzec B (2009). Int J Environ Anal Chem.

[18] Boryło A, Skwarzec B, Olszewski G (2012). J Environ Sci Health A.

[19] Boryło A, Nowicki W, Skwarzec B (2013). Polish J Environ Stud.

[20] Boryło A, Skwarzec B (2011). Radiochim Acta.

[21] Andreou G, Efstathiou M, Pashalidis I (2012). J Radioanal Nucl Chem.

[22] Wang X, Peng G, Yang Y, Wang Y, He T (2012). J Radioanal Nucl Chem.

[23] Skwarzec B (1997). Chem Anal.

[24] Skwarzec B, Namieśnik J, Szefer P (2009). Determination of radionuclides in aquatic environment. Analytical measurement in aquatic environments.

[25] Boryło A (2013). J Radioanal Nucl Chem.

[26] Bonatti E, Fisher D, Joensuu O, Rydel H (1971). Geochim Cosmochim Acta.

[27] Nikołajew DS, Łazariew KF, Korn OP, Drożin WM (1966). Radiochimija.

[28] Sackett WM, Mo T, Spalding RF, Exner ME (1973). A revolution of the marine geochemistry of uranium [w:] Radioactive contamination of the marine environment.

[29] Starik IE, Koljadin LB (1957). Gieochimija.

[30] Skwarzec B (2011) Radionuclides in sediments and benethic organisms. In: Geochemistry of Baltic Sea surface sediments, Państwowy Instytut Geologiczny (red. Sz. Uścinowicz), Warszawa

[31] Skwarzec B, Boryło A, Strumińska D (2002). J Environ Radioact.

[32] Skwarzec B, Boryło A, Strumińska DI (2004). Water Air Soil Pollut.

[33] Skwarzec B, Strumińska DI, Boryło A (2006). Nukl.

[34] Skwarzec B, Strumińska-Parulska DI, Boryło A, Kabat K (2012). J Environ Sci Health A.

[35] Boryło A, Skwarzec B, Fabisiak J (2010). J Radioanal Nucl Chem.

[36] Skwarzec B, Jahnz-Bielawska A, Boryło A (2010). Radiochim Acta.

[37] Skwarzec B, Tuszkowska A, Boryło A (2010). Oceanology.

[38] Skwarzec B, Boryło A, Prucnal M, Strumińska-Parulska D (2010). Polish J Environ Stud.

[39] Mietelski JW (2000). Appl Radiat Isot.

[40] Pietrzak-Flis Z, Suplińska MM, Rosiak L (1997). J Radioanal Nucl Chem.

[41] Martinez-Aguirre A, Garcia-León M, Ivanovich M (1994). Nucl Instrum Methods A.

[42] Periáňez R, Martinez-Aguirre A, Garcia-León M (1996). Appl Radiat Isot.

[43] Boryło A, Nowicki W, Olszewski G, Skwarzec B (2012). J Environ Sci Health A.

[44] Sert E, Uğur A, Özden B, Murat Saç M, Camgöz B (2011). J Environ Radioact.

